# SpvC is a *Salmonella* effector with phosphothreonine lyase activity on host mitogen-activated protein kinases

**DOI:** 10.1111/j.1365-2958.2008.06134.x

**Published:** 2008-03

**Authors:** Piotr Mazurkiewicz, Jerry Thomas, Jessica A Thompson, Mei Liu, Laurence Arbibe, Philippe Sansonetti, David W Holden

**Affiliations:** 1 Centre for Molecular Microbiology and Infection, Imperial College London Armstrong Road, London SW7 2AZ, UK; 2 Technology Facility, Department of Biology, University of York Heslington, York, UK; 3 Unité de Pathogénie Microbienne Moléculaire, INSERM U786, Institut Pasteur 28 Rue du Docteur Roux, 75724 Paris Cedex 15, France

## Abstract

SpvC is encoded by the *Salmonella* virulence plasmid. We have investigated the biochemical function of SpvC and the mechanism by which it is secreted by bacteria and translocated into infected macrophages. We constructed a strain carrying a deletion in *spvC* and showed that the strain is attenuated for systemic virulence in mice. SpvC can be secreted *in vitro* by either the SPI-1 or SPI-2 type III secretion systems. Cell biological and genetic experiments showed that translocation of the protein into the cytosol of macrophages by intracellular bacteria is dependent on the SPI-2 T3SS. Using antibodies specific to phospho-amino acids and mass spectrometry we demonstrate that SpvC has phosphothreonine lyase activity on full-length phospho-Erk (pErk) and a synthetic 13-amino-acid phospho-peptide containing the TXY motif. A *Salmonella* strain expressing *spvC* from a plasmid downregulated cytokine release from infected cells.

## Introduction

*Salmonella enterica* serovar Typhimurium (*S.* Typhimurium) is a facultative intracellular pathogen that causes a self-limiting gastroenteritis in humans and, less commonly, a non-typhoidal bacteremia (Velge *et al*., 2005). *S.* Typhimurium pathogenesis depends upon a large number of virulence proteins. Some of these, called effectors, are injected into the host cell by two specialized protein translocation machineries – type three secretion systems (T3SS). The T3SS encoded within *Salmonella* pathogenicity island 1 (SPI-1) is produced by extracellular bacteria and delivers effectors across the host cell plasma membrane; these are necessary for bacterial internalization and the early stages of *Salmonella*-containing vacuole (SCV) formation (Zhou and Galan, 2001; Schlumberger and Hardt, 2006). The SPI-2-encoded T3SS is activated by internalized *Salmonella* and translocates effectors across the vacuolar membrane. These support bacterial replication in epithelial cells and macrophages, interfere with MHC class II presentation (Mitchell *et al*., 2004) and also induce macrophage motility (Worley *et al*., 2006) and cytotoxicity (Rytkonen *et al*., 2007). Together, SPI-2 T3SS effectors are required for systemic virulence in the murine model of salmonellosis (Shea *et al*., 1996; Waterman and Holden, 2003).

Some *Salmonella* serovars carry plasmids which share a highly conserved locus called the *spv* (*salmonella plasmid virulence*) operon (Boyd and Hartl, 1998). This operon is primarily responsible for the virulence phenotype associated with these plasmids (Gulig *et al*., 1993; Gulig and Doyle, 1993; Chu and Chiu, 2006). It has been suggested that *spv* genes are important for pathogenesis in humans as *spv*-carrying strains dominate among clinical isolates from patients with non-typhoidal bacteremia (Montenegro *et al*., 1991; Fierer *et al*., 1992). *spv* genes are also necessary for full virulence of *S.* Typhimurium in mouse models of systemic infection (Gulig and Doyle, 1993), but their functions are poorly understood. The operon consists of five genes, named *spvR*, *A*, *B*, *C* and *D* (Krause *et al*., 1992). The expression of *spv* genes is strongly induced in intracellular bacteria in tissue culture, in animal infection models and in media mimicking the ion composition and low pH within the SCV (Wilson *et al*., 1997; Wilson and Gulig, 1998; Ygberg *et al*., 2006). The expression of the operon depends on *spvR,* which encodes a positive transcriptional regulator (Fang *et al*., 1991; Krause *et al*., 1992; Grob and Guiney, 1996). *spvA* seems to be dispensable for virulence and its function is unknown (Roudier *et al*., 1992; Wilson and Gulig, 1998). *spvB* encodes an actin-ribosyltransferase, which upon delivery to host cells modifies actin and blocks its polymerization into F-actin filaments (Otto *et al*., 2000; Lesnick *et al*., 2001; Tezcan-Merdol *et al*., 2001). During infections of macrophages *in vitro,* the loss of actin cytoskeleton results in a form of delayed cytotoxicity, manifested by cell detachment and apoptosis of infected cells (Libby *et al*., 2000). The function of *spvD* is unknown but it contributes to virulence of *S.* Typhimurium in mice (Matsui *et al*., 2001).

SpvC shares 63% identity at the amino acid level with OspF of *Shigella flexneri.* Two recent reports demonstrated that OspF can remove phosphate groups from host cell mitogen-activated protein kinases (MAPKs) thereby inactivating them (Arbibe *et al*., 2007; Li *et al*., 2007). OspF interference with MAPK-pathways blocks the activation of a subset of NF-kB-regulated genes resulting in impaired chemokine signalling and compromised recruitment of polymorphonuclear leukocytes to infected tissues (Arbibe *et al*., 2007). OspF removes phosphate groups of Erk and p38 MAPKs by either phosphatase (Arbibe *et al*., 2007) or phosphothreonine lyase (Li *et al*., 2007) activity. Experiments with recombinant SpvC and cells producing SpvC following transfection suggested that it is also capable of inactivating MAPKs (Li *et al*., 2007).

In this study we have investigated the function of SpvC in more detail. We show that SpvC is important for *Salmonella* virulence in mice and that it can be secreted by either the SPI-1 or SPI-2 T3SS. Translocation of SpvC into the cytosol of macrophages is shown to be dependent on the SPI-2 T3SS. Using antibodies specific to phospho-amino acids and mass spectrometry we demonstrate that SpvC has phosphothreonine lyase activity on full-length phospho-Erk (pErk) and a synthetic 13-amino-acid phospho-peptide containing the TXY motif. A *Salmonella* strain expressing *spvC* from a plasmid downregulated cytokine release from infected cells.

## Results

### SpvC is a *Salmonella* T3SS effector

**Fig. 1:**
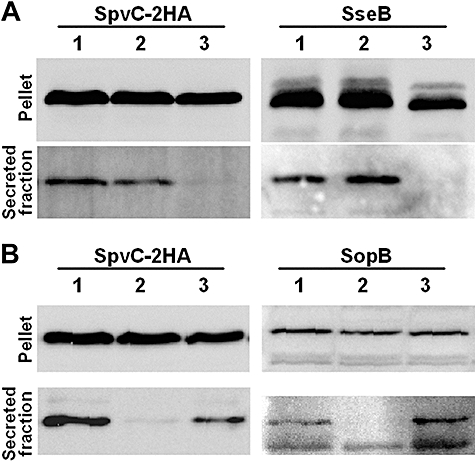
*In vitro* secretion of SpvC-2HA. A. Secretion of double HA-tagged SpvC produced from the virulence plasmid by *Salmonella* strains cultured in low magnesium minimal medium (MgM-MES): 1 –*spvC*-2HA, 2 –*prgHspvC*-2HA, 3 –*ssaVspvC*-2HA. B. Constitutively produced SpvC-2HA is secreted in SPI-1 T3SS-dependent manner by *Salmonella* grown in LB medium: 1 – WT p*spvC*, 2 –*prgH*p*spvC*, 3 –*ssaV*p*spvC*. Samples were probed for SseB and SopB to confirm the activity of SPI-2 and SPI-1 T3SSs respectively.

*spvC* is essential for full virulence of *S.* Typhimurium in mice (Matsui *et al*., 2001) but it has not been established if SpvC functions within the bacterium or is an effector protein translocated into the host cells during or after invasion. To investigate the possibility that SpvC is a secreted effector we used homologous recombination to introduce a double haemagglutinin (2HA) tag at the C-terminus of SpvC on the *Salmonella* virulence plasmid (Uzzau *et al*., 2001). Then we checked production of SpvC-2HA by *Salmonella* grown in Luria–Bertani (LB) or MgM-MES media. SpvC-2HA could only be detected at a very low level in the stationary growth phase in LB medium, but a strong induction was observed during exponential growth phase in MgM-MES (data not shown). Production of SpvC-2HA in MgM-MES medium (which mimics the luminal environment of the SCV and which induces expression of SPI-2 T3SS genes) raised the possibility that SpvC might be translocated by the SPI-2 T3SS. *Salmonella* strains carrying mutations in SPI-1 or SPI-2 genes and expressing SpvC-2HA from the virulence plasmid were grown in MgM-MES medium. Immunoblotting was used to detect HA-tagged protein in bacterial lysates and on the plastic surface of the culture vessels, which adsorbs secreted proteins (Daefler, 1999). Similar amounts of SpvC-2HA were present in lysates from wild-type bacteria and strains carrying mutations in *prgH* and *ssaV* (which inactivate the SPI-1 and SPI-2 T3SSs respectively) (Fig. 1A). SpvC-2HA was also present on the plastic surface of the tubes in which wild-type and *prgH* mutant bacteria were cultured, but it was absent from the surface of the tube containing the *ssaV* mutant strain, indicating that SpvC is secreted in a SPI-2 T3SS-dependent manner (Fig. 1A). To test the activity of the SPI-2 T3SS in the strains used, we examined the secretion of SseB, a component of the SPI-2 T3SS translocon (Beuzon *et al*., 1999). As expected, SseB was detected in secreted fractions from wild-type *Salmonella* and *prgH* mutant bacteria but not from the *ssaV* mutant (Fig. 1A). When SpvC-2HA was overproduced from the constitutive *tet* promoter of pACYC184 (p*spvC*), a SPI-1 T3SS-dependent secretion was detected in SPI-1-inducing conditions (Fig. 1B).

**Fig. 2:**
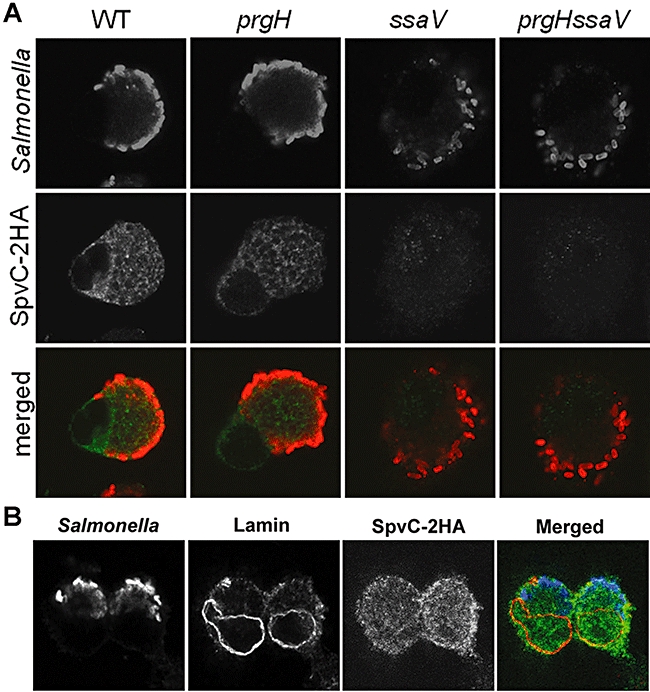
SpvC-2HA is translocated into infected macrophages in SPI-2 T3SS-dependent manner. A. J774 cells were infected for 16 h with *Salmonella* strains carrying pACYC*spvC*-2HA; 10 μg ml^−1^ of a proteasome inhibitor MG132 was added to the culture media for the last 2 h of infection. Samples were fixed and labeled with antibodies to visualize *Salmonella* and SpvC-2HA (red and green, respectively, on merged images). Similar results were observed in infected HeLa cells (data not shown). B. Translocated SpvC-2HA does not accumulate in the nucleus of infected cells. PFA-fixed samples were labeled with antibodies to visualize *Salmonella* (blue), SpvC-2HA (green) and lamin, a nuclear membrane protein (red).

To investigate if SpvC is translocated into infected cells, J774 macrophage-like cells were infected with wild-type *Salmonella* expressing SpvC-2HA from the virulence plasmid. Using antibodies against the 2HA tag we could not detect any translocated SpvC-2HA within infected cells by immunofluorescence microscopy. However, after infection of macrophages with the strain harbouring p*spvC* it was possible to detect translocated SpvC-2HA 16 h post challenge. The amounts of detectable SpvC-2HA increased when infected cells were incubated for 2 h prior to fixation with MG132, a proteasome inhibitor (Fig. 2A). In the majority of examined cells, translocated SpvC-2HA appeared to be distributed evenly in the cytoplasm. These results suggest that relatively small amounts of SpvC are translocated into the cytoplasm of infected cells, where it is prone to degradation. No colocalization with the SCV membrane or accumulation in the nucleus was noted (Fig. 2B). To confirm that translocation of SpvC-2HA is T3SS-dependent we infected J774 cells with *prgH*, *ssaV* and double *prgH ssaV* mutants overexpressing SpvC-2HA. Only infection with the *prgH* mutant led to detectable SpvC-2HA in the cytoplasm of infected cells (Fig. 2A). This result is consistent with that of the *in vitro* secretion experiment, and indicates that a functional SPI-2 T3SS is necessary for translocation of SpvC-2HA.

### Virulence tests

**Fig. 3:**
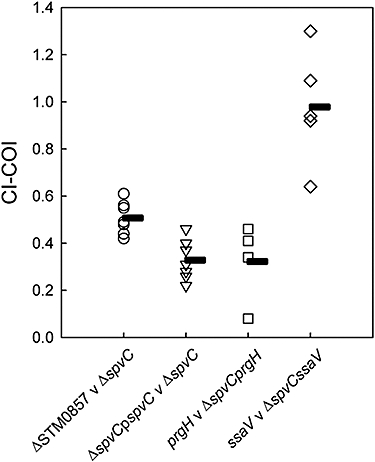
Lack of spvC causes virulence attenuation of *Salmonella* in the mouse model of systemic infection. Canceled-out index (COI) values show that *spvC* function can be genetically linked to the SPI-2 T3SS because the COI of Δ*ssaV* versus Δ*spvC*Δ*ssaV* was statistically significant to the CI of the WT versus the Δ*spvC* strain (*P* < 0.001). Animals were infected for 96 h; horizontal bars indicate the mean values of CI or COI.

To investigate the role of SpvC in virulence, a clean deletion mutant strain (Δ*spvC*) was constructed as described in *Experimental procedures*. Its virulence was tested in mixed infections with a kanamycin-resistant isogenic strain (STM0857) whose virulence level is indistinguishable from that of wild-type *S.* Typhimurium strain 12023 (J. Poh and D.W. Holden, unpubl. data). BALB/c mice were inoculated intraperitoneally with 5 × 10^4^ cfu Δ*spvC* and 5 × 10^4^ cfu of STM0857. After 96 h of infection mice were sacrificed and bacteria were recovered from spleens and plated on LB and LB agar plates supplemented with kanamycin to discriminate the strains. The competitive index (CI) of Δ*spvC* strain was 0.51 (SD ± 0.069) indicating an attenuation in virulence. When mice were coinfected with the Δ*spvC* strain and Δ*spvC* carrying p*spvC*, the CI for Δ*spvC* was 0.33 (SD ± 0.085), showing that the attenuation of the Δ*spvC* strain is due to the loss of SpvC, and that SpvC-2HA is a functional protein. Next, we quantified the attenuation caused by the Δ*spvC* mutation in strains carrying mutations in essential genes for either the SPI-1 (*prgH*) or SPI-2 (*ssaV*) T3SS. Mixed infections with *prgH*Δ*spvC* and *prgH* mutants showed that the lack of *spvC* has an additive effect on the attenuation of *prgH* mutant, indicating that functions of *prgH* and *spvC* are unrelated. By contrast, the *spvC* mutation did not cause any significant additional attenuation to that observed for the *ssaV* mutant. This provides genetic evidence that the functions of *ssaV* and *spvC* are related (Fig. 3). Together with results shown in Figs 1 and 2, these results strongly suggest that SpvC is translocated by the SPI-2 T3SS *in vivo*.

The *spvC* mutant was also compared with the wild-type strain in terms of its ability to replicate inside host cells. In standard assays involving macrophage-like RAW cells (Ruiz-Albert *et al*., 2002), there were no significant differences between the two strains (results not shown).

### Effect of SpvC on Erk activation in infected cells

**Fig. 4:**
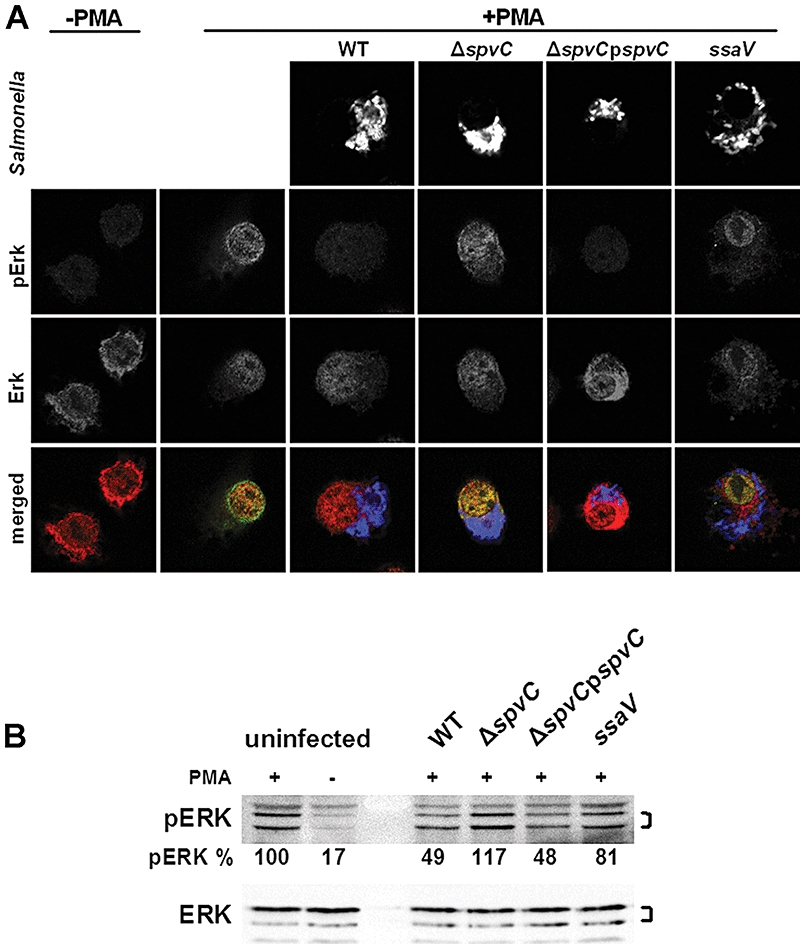
Translocated SpvC prevents Erk phosphorylation upon PMA stimulation. A. J774 cells were infected for 16 h with the indicated *Salmonella* strains (blue in merged images), then Erk activation was triggered by 15 min of incubation with PMA (1 μg ml^−1^). To assess Erk activation total Erk and phosphorylated Erk (pErk) were visualized with antibodies (red and green, respectively, in merged images). Similar results were obtained for HeLa cells (data not shown). B. Western blot analysis of the phosphorylation state of Erk in J774 macrophages infected with different *Salmonella* strains. Lysates of infected cells were probed for pErk and total Erk. Brackets indicate two forms of the kinase detected Erk1 (44 kDa) and Erk2 (42 kDa). pErk bands were quantified using Scion Image software. In both A and B uninfected cells were used as a control for the efficiency of PMA stimulation.

OspF inactivates MAP kinases Erk, p38 (Arbibe *et al*., 2007; Li *et al*., 2007) and possibly JNK (Li *et al*., 2007). As SpvC is translocated into host cells and is 63% identical to OspF, we examined its effect on the activation state of host MAP kinase Erk1/2. J774 cells were infected with different strains of *S.* Typhimurium for 16 h (Fig. 4). Immediately before fixation for immunofluorescence microscopy (Fig. 4A) or cell lysis for Western blotting (Fig. 4B), phorbol-12-myristate 13-acetate (PMA) was added to induce the activation of Erk. This was detected by probing samples with antibodies recognizing double phosphorylated Erk (pErk) on threonine and tyrosine residues of the TXY motif. As shown in Fig. 4, low levels of activated Erk were detected in uninfected, PMA-untreated J774 cells. PMA treatment induced Erk phosphorylation in uninfected control cells, as well as in cells infected with either the Δ*spvC* or *ssaV* mutant. However, infection with wild-type *S.* Typhimurium or the complemented Δ*spvC* strain strongly reduced Erk activation by PMA treatment. These results show that both SpvC and a functional SPI-2 T3SS are necessary for Erk inactivation.

### Purified SpvC inactivates Erk and JNK in vitro and is not sensitive to vanadate inhibition

**Fig. 5:**
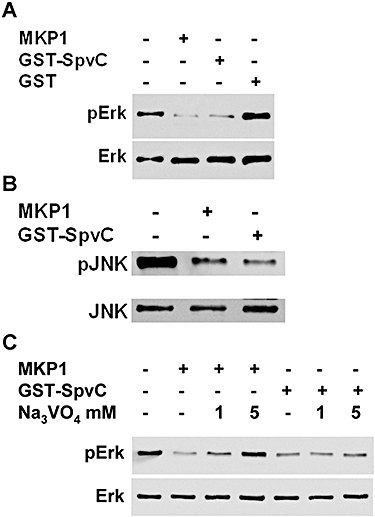
Purified GST–SpvC removes phosphate group(s) from pErk and pJNK *in vitro*. Phosphorylation status of Erk/JNK was determined using an antibody specific for double (threonine and tyrosine) phosphorylated MAP kinases (upper panels in A, B and C). Antibodies detecting MAP kinases regardless of their phosphorylation state were used to check the amounts of pErk/pJNK used (lower panels in A, B and C). A. One hundred nanograms of pErk were incubated with 30 U (1 μg) of MAP-kinase phosphatase 1 (MKP1), 400 ng of GST–SpvC, 400 ng of GST or left untreated as a positive control for anti-pErk antibody. B. One hundred nanograms of pJNK were incubated with 30 U MKP1, 400 ng of GST–SpvC or left untreated as a positive control for anti-pJNK antibody. C. Effect of orthovanadate on the activity of MKP1 and GST–SpvC. Two hundred nanograms of pErk were incubated with 1 μg of MKP1, 200 ng of GST–SpvC and indicated concentrations of sodium orthovanadate.

To establish if SpvC is sufficient for Erk inactivation we purified GST–SpvC (Fig. S1), GST and SpvC-His and used these in *in vitro* dephosphorylation assays with purified, phosphorylated MAP kinases. A dual specificity MAP kinase phosphatase 1 (MKP1) was used as a control for MAP kinase dephosphorylation. Immunoblotting analysis revealed that MKP1 and GST–SpvC can remove phosphate groups from pErk (Fig. 5A). Similar results were obtained when pJNK was incubated with MKP1 and GST–SpvC (Fig. 5B). Next we tested the sensitivity of SpvC to vanadate, a tyrosine phosphatase inhibitor. pErk was incubated with GST–SpvC in the presence or absence of vanadate. As a control, MKP1 (which is susceptible to vanadate inhibition) was used (Charles *et al*., 1993). As expected, dephosphorylation of pErk by MKP1 was inhibited by addition of vanadate, but the activity of SpvC was not (Fig. 5C), indicating that SpvC might not be a typical dual specificity phosphatase.

### SpvC specifically removes phosphate from threonine of the TXY motif

**Fig. 6:**
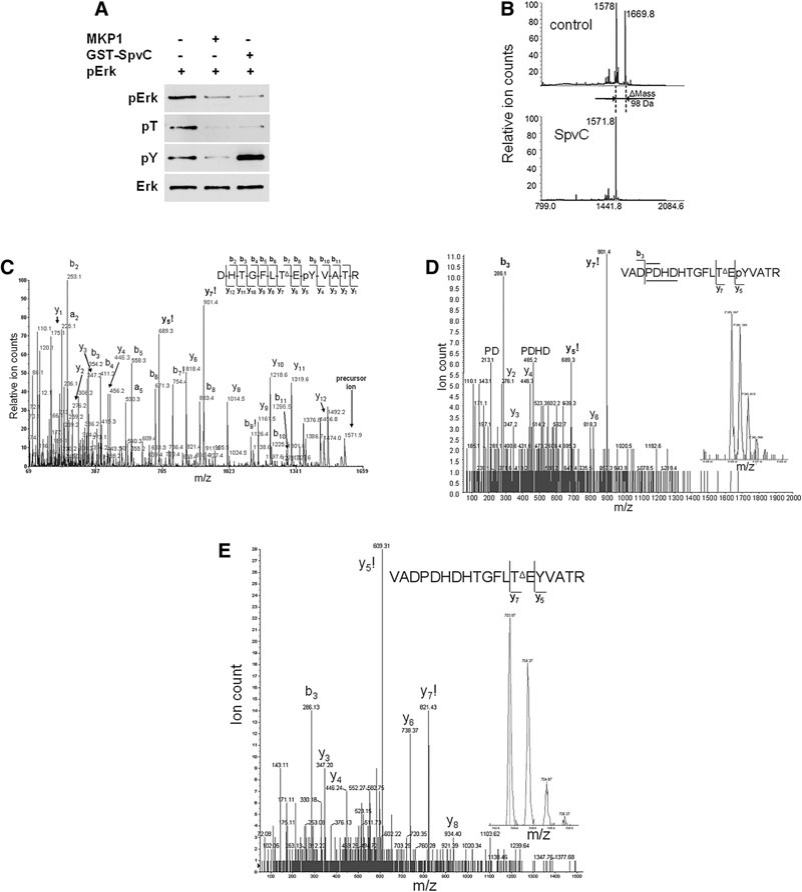
SpvC has phospholyase activity on phospho-threonine but not phospho-tyrosine residues of pErk. A. One hundred nanograms of pErk were incubated with 30 U MKP1, 400 ng of GST–SpvC, or left untreated as a positive control for anti-pErk antibody. Samples were incubated for 1 h at 30°C, and then subjected to SDS-PAGE and blotting. Membranes were probed with antibodies recognizing double phosphorylated Erk (pErk), phosphothreonine residues (pT), phosphotyrosine residues (pY) or Erk regardless of its phosphorylation state (Erk). B. MALDI-TOF spectrum of a synthetic Erk2 diphosphopeptide (DHTGFL-pT-E-pY-VATR) untreated (control) and treated with SpvC. The mass difference of 98 between the diphosphopeptide ([M + H]^+^ at *m/z* 1669.8) and the SpvC-treated product ([M + H]^+^ at *m/z* 1571.8) is consistent with the loss of phosphoric acid HPO3 and H_2_O. C. Tandem mass spectrometric (MALDI-CID-MS/MS) analysis of the precursor at m/z 1571.8 from the SpvC-treated fraction. The b- and y-ions are labelled; those with an exclamation mark indicating ions are crucial to assignment of the sites of phosphorylation (tyrosine-9) and dehydration (threonine-7). All ions included in the diagram are visible in the spectrum. T^Δ^ denotes dehydrothreonine and pY phosphotyrosine. D. CID-MS/MS spectrum of the triply charged precursor ([M + 3H]^3+^) at m/z 736.0 obtained by LC-ES-MS of the tryptic digest of SpvC-treated pErk. The y-ions with an exclamation mark are crucial to assignment of the sites of phosphorylation (tyrosine-15) and dehydration (threonine-13). Double b,y-cleavage ions are indicated by PD and PDHD, and their proposed origins are indicated by horizontal lines above and below the peptide sequence. T^Δ^ denotes dehydrothreonine and pY phosphotyrosine. Inset shows the precursor ion at m/z 736.0 in an LC-MS survey scan recorded at a retention time of 4.96 min. E. CID-MS/MS spectrum of the triply charged precursor ([M + 3H]^3+^) at m/z 703.9 obtained by LC-ES-MS of the tryptic digest of SpvC-treated pErk. The y-ions with an exclamation mark are crucial to assignment of the site of dehydration (threonine-13). T^Δ^ denotes dehydrothreonine. Inset shows the precursor ion at m/z 703.9 in an LC-MS survey scan recorded at a retention time of 5.08 min.

The antibody used above recognizes double-phosphorylated Erk on threonine and tyrosine residues in the conserved activation loop motif TEY. This antibody does not recognize unphosphorylated Erk or Erk phosphorylated on either threonine or tyrosine. Therefore, to establish if SpvC is a dual specificity enzyme or if it acts only on phospho-threonine or phospho-tyrosine residues, we probed MKP1- or SpvC-treated pErk with antibodies recognizing only phospho-threonine or only phospho-tyrosine residues. MKP1-treated pErk lost phosphate groups on both threonine and tyrosine (Fig. 6A). By contrast, SpvC-treated pErk was negative for phospho-threonine labelling but remained positive for phospho-tyrosine labelling. Furthermore, the anti-phosphotyrosine antibody gave a stronger signal from the SpvC-treated pErk sample than from untreated pErk, suggesting that the presence of phosphate group on the threonine residue adjacent to the phospho-tyrosine might reduce binding of anti-phosphotyrosine antibody to double phosphorylated Erk. These results suggest that SpvC is not a dual specificity phosphatase but is either a phosphothreonine-specific phosphatase or, as proposed for OspF (Li *et al*., 2007), a phosphothreonine lyase.

### SpvC is a phosphothreonine lyase

To discriminate between phosphatase and lyase activities we analysed a 13-amino-acid diphosphopeptide [comprising the TEY motif and adjacent amino acids; Erk2(177–189)] after treatment with SpvC, using mass spectrometry. MALDI-TOF mass spectra of Erk2(177–189) before and after treatment with SpvC (Fig. 6B) show that a mass shift of 98 Da occurred as a result of treatment with the enzyme. The monoisotopic [M + H]^+^ ion at *m/z* 1669.8 of the diphosphorylated peptide (theoretical *m/z* 1669.66) was replaced by a product at *m/z* 1571.8, which was consistent with the loss of phosphoric acid (H_3_PO_4_). The loss of phosphoric acid from threonine-7 of the Erk2(177–189) peptide to form dehydrothreonine was confirmed by MALDI-CID-MS/MS of the precursor ion at *m/z* 1571.8 (Fig. 6C). Product ions crucial to the assignment of the site of dephosphorylation (indicated with an exclamation mark) are the b_7_ at *m/z* 754 and y_7_ at *m/z* 901. The mass difference of *m/z* 83 between the y_6_ and y_7_ ions is consistent with dehydrothreonine; threonine at this position would result in a mass difference of *m/z* 101. Phosphorylation at tyrosine-9 was confirmed by the b_9_ ion at *m/z* 1126 and y_5_ at *m/z* 689. The MS/MS spectrum of the dephosphorylated Erk2(177–189) peptide produced by SpvC treatment (Fig. 6C) is very similar to the spectrum of the OspF product obtained by Li *et al*. (2007). The compound giving rise to the ion at *m/z* 1578.0 in the spectrum of the Erk(177–189) peptide preparation could not be identified. Despite being lighter by 91 Da, its MALDI-CID-MS/MS spectrum was almost identical to that of the ion at *m/z* 1669.8 (data not shown). This compound therefore appears to be a peptide that is very similar to the diphosphorylated Erk(177–189) peptide.

Dephosphorylation of p-Erk2 by SpvC was characterized by mass spectrometry of the tryptic peptides produced by in-gel digestion of the protein after SpvC treatment and SDS-PAGE. The tryptic peptide VADPDHDHTGFLTEYVATR with a single phosphorylation site and a dehydrothreonine residue, which would be produced from the p-Erk2 by SpvC acting as lyase, has an expected [M + H]^+^ monoisotopic mass of 2206. An ion with this mass was observed in the MALDI-TOF spectrum of the tryptic digest of SpvC-treated p-Erk2 (data not shown), but its intensity was not sufficient for selection as a precursor ion for MS/MS. The same peptide was detected as the triply charged [M + 3H]^3+^ ion at *m/z* 736 during LC-ES-MS (Fig. 6D, inset). The CID-MS/MS spectrum of the triply charged ion at *m/z* 736 contains product ions that are consistent with the sequence VADPDHDHTGFLT^Δ^EpYVATR, where T^Δ^ indicates dehydrothreonine and pY indicates phosphotyrosine in the sequence. Ions crucial to the sequence assignment are present, in particular the y_5_ and y_7_ ions at *m/z* 689 and 901 respectively. The proposed double-cleavage ions at *m/z* 213 and 465 are entirely consistent with the peptide sequence and the enhanced cleavage normally observed at prolyl and aspartatyl residues (Wysocki *et al*., 2000). In addition to the tyrosine-phosphorylated tryptic peptide containing dehydrothreonine, the non-phosphorylated version was also observed by LC-ES-MS (Fig. 6E). The presence of this non-phosphorylated peptide product indicates that the p-Erk2 substrate was not completely phosphorylated at tyrosine-412, as well as providing additional evidence for the removal of phosphoric acid from threonine-410 by SpvC.

### Effect of SpvC on proinflammatory immune responses

**Fig. 7:**
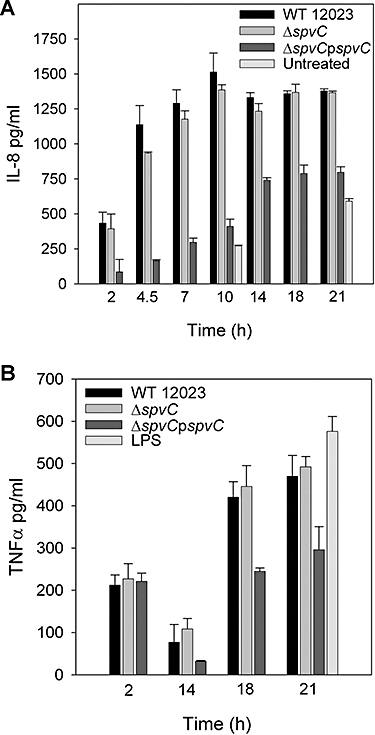
Effect of *Salmonella* on cytokine release by infected cells. ELISA was used to measure amounts of IL-8 released by infected HeLa cells (A) and TNF-α released by J774 macrophages (B) into the growth media at the indicated time points. Similar infection efficiencies was confirmed by microscopy in every experiment. *P*-values were calculated for cytokine levels from cells infected with the Δ*spvC*p*spvC* strain compared with cells infected with WT bacteria were significantly different (> 0.05) at all time points tested for A, and at 14, 18 and 21 h for B.

The action of OspF on MAPKs affects histone modification and chromatin accessibility of NF-κB at the IL-8 promoter, and negatively regulates neutrophil recruitment in infected tissues (Arbibe *et al*., 2007). We therefore measured secretion of IL-8 from HeLa cells at different times after infection with bacterial strains. Infection with wild-type bacteria induced IL-8 production but no increased production could be detected in cells infected with the Δ*spvC* mutant (Fig. 7A). However, cells infected with the Δ*spvC*p*spvC* strain showed significantly less IL-8 production compared with the wild-type strain at all time points tested (*P* ≤ 0.05 for all time points) (Fig. 7A). As TNF-α production following stimulation of TLR-4 by LPS is MAPK-dependent (van der Bruggen *et al*., 1999), we also examined production of this cytokine by murine J774 macrophages. Time points for sampling were chosen to coincide with detectable SpvC translocation into host cells. Again, no significant differences were detected between cells infected with the wild-type or *spvC* mutant, but cells overexpressing SpvC produced less TNF-α at 14, 18 and 21 h post uptake (Fig. 7B). We also infected BALB/c mice with either the wild-type, *spvC* mutant or Δ*spvC*p*spvC* strains. At 4, 24 and 72 h post inoculation, mouse sera were recovered and analysed for IL-6, IL-10, MCP-1, INF-γ, TNF-α and IL12p70 by ELISA. No statistically significant differences were detected between mice infected with these strains (data not shown).

## Discussion

Phylogenetic analysis suggests that the *spv* operon is widely distributed throughout the subspecies of *Salmonella enterica.* It is usually carried on the large *Salmonella* virulence plasmid, but in some serovars is integrated into the chromosome (Boyd and Hartl, 1998). The best characterized Spv protein is SpvB (which is encoded immediately upstream of *spvC* in the operon). SpvB ADP-ribosylates actin and is associated with cytotoxic activity in infected host cells (Otto *et al*., 2000; Tezcan-Merdol *et al*., 2001; Browne *et al*., 2002). SpvB must therefore be translocated into host cells from extracellular or intracellular bacteria. Its cytopathic effects seem to be dependent on a functional SPI-2 T3SS (Browne *et al*., 2002), but paradoxically its secretion into the culture medium *in vitro* was reported to be independent of either the SPI-1 or SPI-2 T3SSs (Gotoh *et al*., 2003).

Until recently little has been known about the function of SpvC, apart from its requirement for virulence in mice (Matsui *et al*., 2001). Using an epitope-tagged version produced from its own promoter in the virulence plasmid, we have shown here that SpvC can be secreted into the culture medium in either a SPI-1- or SPI-2-dependent manner, according to which secretion system is activated by bacterial growth conditions. However, we were unable to detect translocation of the protein by immunofluorescence microscopy following its normal production in infected cells. Only when SpvC was produced from a multicopy plasmid in infected cells that had been treated with a proteasome inhibitor could its SPI-2 T3SS-dependent translocation be detected clearly.

Although SpvC was not observed in infected macrophages following expression from its own promoter, phospho-Erk was detected in the cytoplasm of cells infected with either the *spvC* and SPI-2 T3SS null mutant, but not in cells infected with the wild-type or *spvC*-complemented strain (Fig. 4). As SpvC removes phosphate groups from Erk, this provides strong indirect evidence for SPI-2-dependent translocation of SpvC by wild-type bacteria. Furthermore, virulence tests with single and double mutants demonstrated that the attenuation caused by mutation of *spvC* is SPI-2-dependent, indicating that SpvC uses this secretion system *in vivo*. This contrasts with the results of an earlier study in which a polar *spvA* mutation had an additive effect on virulence attenuation of a SPI-2 null mutant (Shea *et al*., 1996). It is possible that other protein(s) encoded by the operon might be functionally independent of the SPI-2 T3SS, but further work on secretion and translocation of the Spv proteins is necessary to clarify this issue. Taken together, the results of this study provide strong evidence that during intracellular growth of bacteria, SpvC is translocated into the cytosol of macrophages in relatively small amounts through the SPI-2 T3SS. Whether or not it is also translocated along with SPI-1 T3SS effectors during invasion of epithelial cells remains to be determined.

There are approximately 31 genes in SPI-2 that encode the secretion apparatus, translocon, chaperones and at least two effectors. These, together with genes for several other effectors located outside SPI-2, are strongly induced following uptake of bacteria by host cells and require the SPI-2-encoded SsrA-B two-component system for their expression (Cirillo *et al*., 1998). The *spv* operon is known to be induced during intracellular growth in macrophages (Rhen *et al*., 1993; Eriksson *et al*., 2003), but to our knowledge there is no evidence indicating that its expression is dependent on the SsrA-B two-component system. Indeed, we found that RNA levels for *spvA, B, C and D* were similar in wild-type and *ssrA* mutant bacteria that had been grown in conditions that result in strong expression of the SsrA-SsrB regulon (Rytkonen *et al*., 2007; P. Mazurkiewicz and D.W. Holden, unpubl. results). SlrP and SspH 1 are two other effectors that can be translocated by either the SPI-1 or SPI-2 T3SSs, and neither seems to be regulated by SsrA-B (Miao and Miller, 2000). Therefore, the repertoire of SPI-2 T3SS effectors is not restricted to the SsrA-B regulon, and the total number of SPI-2 effectors could be significantly larger than previously anticipated.

A purified GST–SpvC fusion protein removed phosphate groups from phospho-Erk and phospho-JNK *in vitro*, and its insensitivity to vanadate suggested that it is not a typical tyrosine phosphatase. Subsequent mass spectrometry analysis of both full-length Erk and a synthetic phosphopeptide established that it acts as a phosphothreonine lysase, as has been shown for OspF of *S. flexneri* (Li *et al*., 2007). A potential catalytic mechanism has been proposed recently, based on an analysis of the crystal structure of SpvC (Zhu *et al*., 2007). This form of phosphate removal appears to be irreversible, which might help to explain why we could detect the effects of SpvC activity on levels of phospho-Erk in infected cells, but not the bacterial effector itself.

The action of OspF on MAP kinases inhibits immune responses by impairing the formation of histone H3-phosphorylated and NF-κB-regulated promoters (Arbibe *et al*., 2007). This is associated with a pronounced decrease in IL-8 production by infected cells and reduced recruitment of polymorphonuclear leucocytes to infected tissues (Arbibe *et al*., 2007). It would appear that SpvC performs a similar function in *Salmonella*. However, while SpvC is clearly a phosphothreonine lyase, it is possible that its substrate specificity and/or overall physiological effects are different to OspF. The proteins share 63% amino acid identity, and this could allow differential specificity towards MAP kinases. In support of this, Arbibe *et al*. (2007) reported that OspF is active towards Erk and p38, but not JNK, while SpvC acts on Erk, p38 and JNK (Li *et al*., 2007; and this work). A second possible means by which the proteins could exert different activities results from the spatio-temporal context in which they are produced: OspF is injected by extracellular bacteria shortly after bacterial contact with host cells and localizes to the nucleus, while SpvC is translocated into the host cytoplasm relatively late in the infective process from intracellular bacteria. Furthermore, whereas OspF accounted for strong suppression of IL-8 transcription and secretion between 2 and 5 h following bacterial invasion of Caco-2 cells, we did not observe a significant difference between the wild-type or *spvC* mutant strains in the levels of IL-8 produced from HeLa cells, TNF-α released from mouse macrophages (despite the clear difference in levels of phospho-Erk in infected cells) or six cytokines that were tested in sera from infected mice. Nevertheless, it seems likely that SpvC does inhibit proinflammatory responses, because the complemented mutant overexpressing SpvC caused a strong reduction of IL-8 and TNF-α production in tissue cultured cells. It is possible that the effects of SpvC produced by wild-type bacteria are masked by other immune-modulating bacterial molecules present in the infection assays. *In vivo*, SpvC could help suppress localized proinflammatory responses at infection foci in the spleen and liver, and thereby facilitate bacterial growth. This would be consistent with our finding that the *spvC* mutant does not have a replication defect in tissue cultured macrophages, but is attenuated in the mouse model of systemic infection.

## Experimental procedures

### Bacterial strains and growth conditions

All bacterial strains used in this study are listed in Table S1. Strains were grown in LB medium or a medium based on magnesium minimal medium MES (MgM-MES), containing 170 mM 2-[*N*-morpholino]ethane-sulphonic acid at pH 5.0, 5 mM KCl, 7.5 mM (NH_4_)_2_SO_4_, 0.5 mM K_2_SO_4_, 1 mM KH_2_PO_4_, 8 μM MgCl_2_, 38 mM glycerol and 0.1% casamino acids supplemented with appropriate antibiotics when necessary (For *E. coli*: ampicilin 100 μg ml^−1^, kanamycin 50 μg ml^−1^, chloramphenicol 50 μg ml^−1^; for *Salmonella*: ampicilin 50 μg ml^−1^, kanamycin 50 μg ml^−1^, chloramphenicol 50 μg ml^−1^ or tetracycline 12.5 μg ml^−1^), at 37°C with aeration if not stated differently.

### Construction of the spvC mutant strains and HA-tagging of spvC on the virulence plasmid

Deletion of the *spvC* gene was performed by the method of Datsenko and Wanner (2000), using primers Del-spvC-F and Del-spvC-R (Table S2) to amplify the Cm^r^ and Kn^r^ cassettes from pSU314 or pSU315 respectively. The resulting PCR product was integrated into the chromosome of *S.* Typhimurium expressing the λ red recombinase from pKD46. To excise the resistance marker the Δ*spvC*^Kn^ strain was transformed with pCP20 helper plasmid expressing the FLP recombinase (Cherepanov and Wackernagel, 1995). Deletion of the *spvC* gene was confirmed by PCR using primers Del-F and Del-R which flank the deleted region. Virulence plasmid-encoded *spvC* was double HA-tagged using the procedure described by Uzzau *et al*. (2001). The Cm^r^ and Kn^r^ cassettes were amplified from pSU314 or pSU315, respectively, using primers spvC-F and spvC-R and the resulting PCR products were integrated into virulence plasmid of various *S.* Typhimurium strains (Table S1).

### Construction of plasmids

The bacterial plasmids used in this study are listed in Table S1. To obtain pACYC*spvC*-2HA the double-HA-tagged *spvC* was amplified from the virulence plasmid of *spvC*-2HA strain using pacyc-F and pacycR2HA-C primers; the PCR product was cloned into pACYC184 EcoRV and SalI sites. To amplify *spvC* for cloning into BamHI and EcoRI sites of pGEX4T2, primers pGEXspvC-F and pGEXspvC-R were used. To amplify *spvC* for cloning into NdeI and XhoI sites of pET22b, primers spvC-his-F and spvC-his-R were used. Obtained plasmids were verified by sequencing.

### Cell culture

HeLa (93021013) and J774 (91051511) cells were obtained from the European Collection of Cell Cultures, Salisbury, UK. Cells were grown in DMEM (Gibco, Carlsbad, CA) supplemented with 10% FCS at 37°C in 5% CO_2_.

### Antibodies

Anti-*Salmonella* goat polyclonal antibody CSA-1 (Kirkegaard and Perry Laboratories, Gaithersburg, MD) was used at a dilution of 1:200. Anti-HA mouse monoclonal antibody (HA.11; Covance) was used at a dilution of 1:200 (immunofluorescence microscopy) and 1:1000 (Western blot). Anti-SseB rabbit serum (Beuzon *et al*., 1999) was used at a dilution of 1:5000 for Western blot. Mouse monoclonal anti-pErk antibody (M8159, Sigma) was used at a dilution of 1:200 (immunofluorescence) and 1:1000 (Western blot). Rabbit polyclonal anti-Erk antibody (06–182, Upstate) was used at a dilution of 1:200 (immunofluorescence) and 1:1000 (Western blot). Cy2- and Rhodamine red X (RRX)-conjugated donkey anti-goat, anti-mouse or anti-rabbit antibodies (Jackson Immunoresearch Laboratories) were used for immunofluorescence at a dilution of 1:200 and 1:400 for Cy5-conjugated antibody. Anti-mouse and anti-rabbit HRP (Amersham Pharmacia Biosciences) were used at a dilution of 1:10 000 for Western blot analysis.

### Mice infections

Female BALB/c mice (B and K Universal, UK) that were 7–12 weeks old were used in accordance with UK Home Office regulations. To prepare the inocula, bacteria were first grown overnight in LB broth and then subcultured at a dilution of 1:100 for a further 2 h. Cultures were diluted to a concentration of 2.5 × 10^4^ cfu ml^−1^ in physiological saline and mixed for intraperitoneal inoculation (0.2 ml per mouse). Viable bacteria in inocula were quantified by dilution and plating onto LB agar plates with appropriate antibiotics to distinguish between strains. Mice were sacrificed at various times post inoculation. The spleens were removed aseptically and homogenized in distilled water by mechanical disruption in a Colworth stomacher. Serial dilutions were plated on LB agar for cfu enumeration. Strains were distinguished by differential counting or replica plating on antibiotic-supplemented plates.

For each mouse, the CI or cancelled-out index (COI) was calculated by dividing the output ratio (i.e. wild-type versus mutant *a*, or mutant *a* versus mutant *ab*) divided by the input ratio. The CI values were used to calculate means and in statistical analyses. Student's *t*-test was used to analyse the COI of the single-mutant versus double-mutant strains (i.e. mutant *b* versus mutant *ab*) with the null hypothesis that the mean COI was equal to the CI of the wild-type versus the single mutant (i.e. wild-type versus mutant *a*).

### Infection of epithelial cells and macrophages

Infection of HeLa cells and macrophages was performed as described (Beuzon *et al*., 2000; Ruiz-Albert *et al*., 2002). Cells were fixed with paraformaldehyde, permeabilized and incubated with antibodies as described (Beuzon *et al*., 2000). Labelled cells were analysed by using a fluorescence microscope (BX50; Olympus) or a confocal laser scanning microscope (LSM510; Zeiss).

### ELISA

HeLa cells or J774 macrophages were seeded at a density of 10^5^ cells per well in 24-well Plate 24 h before challenge with various *Salmonella* strains. At different time points after challenge plates were centrifuged at 400 *g* to settle down non-adherent cells. Supernatants were transferred into clean tubes and stored at −80°C until the assay was performed. Purified LPS (Sigma, St Louis, MO) at 1 μg ml^−1^ was used as a positive control stimulus for TNF-α production by J774 macrophages. TNF-α and IL-8 levels were measured using sandwich ELISA according to manufacturer's recommendations (Diaclone, Stamford, CT).

### Measurement of cytokines in mouse sera

Blood samples were collected at various time points after bacterial challenge from intraperitoneally infected mice. Animals were sacrificed and spleens were isolated for enumeration of bacteria. Blood was allowed to clot for 4 h at room temperature and then overnight at 4°C. The clot was separated by centrifugation and sera were stored at −80°C until used for measurements. BD™ Cytometric Bead Array (BD™ CBA, BD Biosciences, San Diego, CA) specific for IL-6, IL-10, IL-12p70, MCP-1, IFN-γ and TNF-α was used to test the sera. Measurements were performed according to manufacturer's recommendations using BD FACSCalibur™ flow cytometer.

### Protein secretion assay

To analyse protein production and secretion in SPI-2 T3SS inducing conditions, *Salmonella* strains were grown overnight in MgM-MES medium. Overnight cultures were subcultured 1:33 into a tube containing 5 ml of fresh growth medium, and bacteria were allowed to grow for a further 6 h. To ensure that protein from equal numbers of cells was analysed, in all experiments protein samples were adjusted to OD_600_ values such that a volume corresponding to 1 ml of a culture of OD_600_ 0.5 was taken up in 100 μl of protein-denaturing buffer for gel electrophoresis. The secreted and total bacterial fractions were prepared as described before (Yu *et al*., 2002).

### Protein purification

Plasmids for production of GST and GST fusion SpvC (GST–SpvC) were transformed into *E. coli* TOP10 cells. Proteins were induced by using IPTG (0.5 mM), and cells were lysed using a French press. Samples were centrifuged at 38 000 *g* for 45 min, and supernatants were incubated with Glutathione Sepharose 4B beads (Amersham Pharmacia Biosciences) at 4°C. The beads were washed extensively with wash buffer (50 mM Tris-Cl, pH 8.0, 100 mM NaCl, 1 mM DTT) and eluted in the same buffer containing 10 mM glutathione. Samples were dialysed against 50 mM Tris-Cl, pH 7.4, 50 mM NaCl. To dialysed samples, 20% glycerol was added and aliquots were stored at −20°C. For purification of six histidine-tagged SpvC, pET22b*spvC*-His was introduced to *E. coli* BL21. Proteins were induced by using IPTG (0.5 mM), and cells were lysed using a French press. Samples were centrifuged at 38 000 *g* for 45 min, and supernatant was incubated with Ni-NTA Agarose (Qiagen). The beads were washed with wash buffer (20 mM Tris-Cl, 300 mM NaCl, 20 mM imidazole, 10% glycerol, pH 8.0) and SpvC-His was eluted with elution buffer (20 mM Tris-Cl, 150 mM NaCl, 250 mM imidazole, 10% glycerol, pH 7.0). Samples were dialysed in 50 mM Tris-Cl, pH 7.4, 50 mM NaCl, 10% glycerol and stored at −20°C. Protein concentration was determined using Bio-Rad RC-DC assay with bovine serum albumin as a standard. Protein purity was confirmed through analysis of samples by SDS-PAGE (Fig. S1).

### Dephosphorylation assay

For *in vitro* dephosphorylation reactions 100 ng of GST-pErk2 (pErk) (14–173, Upstate) was incubated with 30 U (1 μg) of MAP-kinase phosphatase 1 (MKP1) 14–391, Upstate, 400 ng of GST–SpvC, 400 ng of GST or left untreated as a positive control for Erk phosphorylation in 100 mM TRIS-Cl pH 7.0, 50 mM NaCl (DB buffer) for 30 min at 30°C. The reactions were stopped by boiling samples in SDS-PAGE sample buffer. For orthovanadate inhibition, an appropriate amount of activated Na_3_VO_4_ was added to the DB buffer. The phosphorylation state of Erk was assessed by Western blot analysis. For mass spectrometry pErk or pErk-derived phosphopeptide (1 μg) were incubated with 400 ng of SpvC-His for 1 h at 30°C in DB buffer. The mixture of pErk and SpvC was separated on a precast Bio-Rad Tris-Glycine acrylamide gel and the band corresponding to pErk was then excised and used for in-gel tryptic digestion. The pErk-derived peptide was used directly for mass spectrometry analysis.

### In-gel tryptic digestion

In-gel tryptic digestion of SDS-PAGE bands was performed after reduction with DTT and S-carbamidomethylation with iodoacetamide. Gel pieces were washed three times with 50% (v/v) aqueous acetonitrile containing 25 mM ammonium bicarbonate and dried in a vacuum concentrator for 30 min. Sequencing-grade, modified porcine trypsin (Promega) was dissolved in the 50 mM acetic acid supplied by the manufacturer, and then diluted fivefold by adding 25 mM ammonium bicarbonate to give a final trypsin concentration of 0.02 μg μl^−1^. Gel pieces were rehydrated by adding 10 μl of trypsin solution, and after 30 min enough trypsin solution was added to cover the gel pieces. Digests were incubated overnight at 37°C.

### MALDI-TOF mass spectrometry

A 0.5 μl aliquot of peptide or tryptic digest was applied directly to a MALDI target plate, followed immediately by an equal volume of a freshly prepared 5 mg ml^−1^ solution of α-cyano-4-hydroxycinnamic acid (Sigma, Poole, UK) in 50% aqueous (v/v) acetonitrile containing 0.1% TFA (v/v). Positive ion mass spectra were obtained using an Applied Biosystems 4700 Proteomics Analyzer (Applied Biosystems, Foster City, CA, USA) in reflectron mode. MS spectra were acquired with a total of 1000 laser pulses over a mass range of *m/z* 800–4000 with a focus mass of *m/z* 1500. CID-MS/MS spectra were acquired using a collision energy of 1 kV with air as the gas at a Source 2 pressure of about 1 × 10^−6^ torr. The precursor mass window was set to a relative resolution of 50, and the metastable suppressor was enabled. MS/MS spectra were baseline-subtracted (peak width 50) and smoothed (Savitsky-Golay with three points across a peak and polynomial order 4) Peak detection used a minimum S/N of 5, local noise window of 50 *m/z*, and minimum peak width of 2.9 bins.

### LC-electrospray (ES) mass spectrometry

Peptide separations were achieved using a 5 cm polystyrene-divinlybenzene (PS-DVB) monolithic column (100 μm inside diameter) and UltiMate capillary HPLC system (Dionex, Sunnyvale, CA, USA) with the column at 60°C. Samples (3 μl) were injected directly onto the column and eluted with a linear gradient of acetonitrile in 0.1% formic acid from 2 to 50% in 10 min at a flow rate of 1.1 μl min^−1^. Peptides eluting from the column were analysed using a QSTAR Pulsar i hybrid mass spectrometer (Applied Biosystems, Foster City, CA, USA) with Analyst QS software and fitted with a modified MicroIonSpray source. The mass spectrometer was operated in information-dependent acquisition mode with an MS survey scan of 1 s and two 0.5 s MS/MS spectra acquired per experiment. Precursor masses were excluded for 120 s after selection, and the collision offset was selected automatically.

## Supplementary material

This material is available as part of the online article from: http://www.blackwell-synergy.com/doi/abs/10.1111/j.1365-2958.2008.06134.x

(This link will take you to the article abstract).
