# Supplementary material for: SpvC is a Salmonella effector with phosphothreonine lyase activity on host mitogen-activated protein kinases
Source: Mol Microbiol. 2008 Mar;67(6):1371–83. doi: 10.1111/j.1365-2958.2008.06134.x (PMC2268955; doi:10.1111/j.1365-2958.2008.06134.x)

SUPPLEMENTARY MATERIAL

Table S1. Bacterial strains and plasmids used in this study

Name	Description	Reference
Strains		
S. Typhimurium		
12023s	Wild type	NTCC[a]
ssaV	Strain HH109 (12023s *ssaV*::*aphT*)	Deiwick *et al.,* (1998)
prgH	Strain HH124 (12023s *prgH*020::Tn5lacZY)	Beuzon *et al.,* (2001)
prgH ssaV	Strain HH193 (12023s *ssaV*::*aphT* *prgH*020::Tn5lacZY)	Beuzon *et al.,* (2001)
Δ*spvC*^{Kn}	12023s Δ*spvC*::Kn^r	This study
Δ*spvC*	as Δ*spvC*^{Kn}, but with excised Kn^r cassette (Kn^s)	This study
Δ*spvC* p*spvC*	Δ*spvC* harboring pACYC*spvC*-2HA	This study
spvC-2HA	12023s *spvC*-2HA::Kn^r	This study
*ssaV*Δ*spvC*	as HH109, Δ*spvC*::Cm^r	This study
*prgH*Δ*spvC*	as HH124, Δ*spvC*::Kn^r	This study
ssaVspvC-2HA	as HH109, *spvC*-2HA::Cm^r	This study
prgHspvC-2HA	as HH124, *spvC*-2HA::Kn^r	This study
Plasmids		
pSU314	Rep$_{R6K}$ Ap^r *2HA* FRT Cm^r FRT	Uzzau *et al.,* (2001)
pSU315	Rep$_{R6K}$ Ap^r *2HA* FRT Kn^r FRT	Uzzau *et al.,* (2001)
pKD46	Rep$_{pSC101}$^{ts} Ap^r p$_{araBAD}$ γ β exo	Datsenko and Wanner (2000)
pCP20	*FLP*$^+$ λ p_R Rep^{ts} Ap^r Cm^r	Cherepanov and Wackernagel (1995)
pACYC184	Rep$_{p15A}$ Cm^r Tet^r low copy number vector	Chang and Cohen (1978)
p*spvC*	pACYC184 with C-terminal, 2-HA-tagged *spvC* cloned into *EcoR*V and *Sal*I sites, Cm^r	This study
pGEX4T2	Vector for expression of GST fusion proteins	*Amersham Biosciences*
pGEX*spvC*	pGEX4T2 with *spvC* cloned between *BamH*I and *EcoR*I sites	This study
pET22b	Vector for expression of His-tagged proteins	*Novagen, Inc.*
pET*spvC*-His	pET22b with *spvC* cloned between *Nde*I and *Xho*I sites, with C-terminal 6-His tag and thrombin cleavage site allowing removal of the tag	This study

[a]NTCC, National Type Culture Collection

References:

Deiwick, J., Nikolaus, T., Shea, J.E., Gleeson, C., Holden, D.W. and Hensel, M. (1998) Mutations in *Salmonella* pathogenicity island 2 (SPI2) genes affecting transcription of SPI1 genes and resistance to antimicrobial agents. *J Bacteriol* **180**: 4775-4780.

Beuzón, C.R., Unsworth, K.E. and Holden, D.W. (2001) In Vivo Genetic Analysis Indicates That PhoP-PhoQ and the *Salmonella* Pathogenicity Island 2 Type III Secretion System Contribute Independently to *Salmonella enterica* Serovar Typhimurium Virulence. *Infect Immun* **69**: 7254-7261.

Uzzau, S., Figueroa-Bossi, N., Rubino, S., and Bossi, L. (2001) Epitope tagging of chromosomal genes in *Salmonella*. *Proc Natl Acad Sci USA* **98**: 15264-15269.

Datsenko, K.A., and Wanner, B.L. (2000) One-step inactivation of chromosomal genes in *Escherichia coli* K-12 using PCR products. *Proc Natl Acad Sci USA* **97**: 6640-6645.

Cherepanov, P.P., and Wackernagel, W. (1995) Gene disruption in *Escherichia coli*: TcR and KmR cassettes with the option of Flp-catalyzed excision of the antibiotic-resistance determinant. *Gene* **158**: 9-14.

Chang, A.C., and Cohen, S.N. (1978) Construction and characterization of amplifiable multicopy DNA cloning vehicles derived from the P15A cryptic miniplasmid. *J. Bacteriol.* **134**: 1141-1156.

Table S2. Primers

Primer	*Sequence (5'-3')*	*Description*
spvC-F	GGCTTTACGTGAGGAACCGTTTTATCGTTTGATGACAGAGTATCCGTATGATGTGCCGGACTATGCGTATCCGTATGATGTTCCTGATTATGCTAGCCTCTAGTAA	For 2HA tagging of SpvC on the virulence plasmid by insertion of Cm^r or Km^r cassette amplified from pSU314 or pSU315, respectively, upstream of *spvC*
spvC-R	CGCTCTTGCTTATGTAATTTTCACCATTACAGTGCGGACCATATGAATATCCTCCTTAGT	
pacycF	GGGGGATATCTAA GGAGATTTCCCATGCCC	For cloning *spvC-2HA* from the virulence plasmid into pACYC184 with an C-terminal 2HA-tag using *EcoR*V and *Sal*I sites
pacycR2HA-C	CGCCCGTCGAC TTACTAGAGGCTAGCATAATC	
Del spvC-F	GGCGATATATCCATATCGCAAAGGAGATTTCCCATGCCTATCCGTATGATGTTCCTGAT	Primers to amplify Cm^r or Km^r cassette from pSU314 or pSU315, respectively, for making deletion of *spvC*
Del spvC-R	AACGGCGTTTACTGTTCCGTTGCTCCCCAAACCCATACCATATGAATATCCTCCTTAG	
Del-F	GAGAATTTATATCTAATAATATG	Primers for confirmation of *spvC* deletion
Del-R	GCATTTAAAATAGCTGTTTAAC	
pGEXspvC-F	CGCCGGAT CC ATGCCCATAAATAGGCC	For cloning *spvC* into pGEX4T2 with a N-terminal GST
pGEXspvC-R	CGCGCGAATTCTTAC TA CTCTGTCATCAAACGATAAA	
spvC-His-F	GCGCGACATATGCCCATAAATAGGCCTAATC	For cloning *spvC* into pET22b with a C-terminal 6-His tag and thrombin cleavage site
spvC-His-R	GCGTGCTCGAGGCTGCCGCGCGGCACCAGCTCTGTCATCAAACGATAAAACGG	

Figure S1. SDS-PAGE and Coomassie Blue staining of purified GST-SpvC (left lane) and GST (right lane) used in *in vitro* assays in this work. Middle lane contains molecular size markers (BioRad).

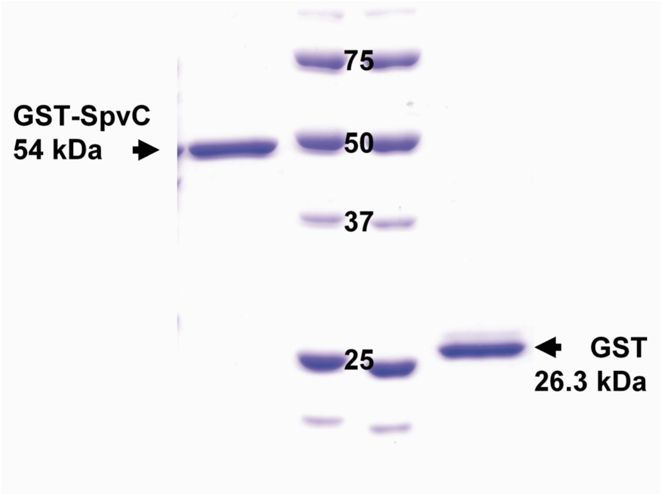

Supplement: Supplementary file 1 [file mmi0067-1371-SD1.pdf]
